# Interaction Between Age and the NOTCH3 p.R544C Variant in Determining Ischemic Stroke Risk: Insights from the Taiwan Biobank Study of 8,588 Patients

**DOI:** 10.7150/ijms.132147

**Published:** 2026-09-24

**Authors:** Yi-Ching Liaw, Oswald Ndi Nfor, Che-Hong Chen, Shu-Yi Hsu, Yi-Chia Liaw, Yung-Po Liaw

**Affiliations:** 1 Department of Nutrition, China Medical University, Taichung 40678, Taiwan.; 2 Department of Nutrition, Chung Shan Medical University, Taichung 40201, Taiwan.; 3 Department of Public Health and Institute of Public Health, Chung Shan Medical University, Taichung, Taiwan.; 4 Department of Public Health, College of Public Health, China Medical University, 406040, Taichung, Taiwan.; 5 Department of Chemical and Systems Biology, Stanford University School of Medicine, Stanford, CA 94305, USA.; 6 Institute of Clinical Medicine, National Yang Ming Chiao Tung University, Taipei, Taiwan.; 7 Division of Neurology, Department of Internal Medicine, National Yang Ming Chiao Tung University Hospital, Yilan, Taiwan.; 8 Department of Medical Research, China Medical University Hospital, China Medical University, Taichung City, Taiwan.

**Keywords:** ischemic stroke, *NOTCH3* rs201118034 p.R544C variant, age, Taiwan Biobank, National Health Insurance Research Database, interaction

## Abstract

Genetic susceptibility to ischemic stroke may vary across the life course, yet the extent to which aging modifies the effect of specific high-risk variants remains incompletely understood. The *NOTCH3* rs201118034 (p.R544C) variant, a CADASIL-related mutation with relatively high prevalence in East Asian populations, has been associated with cerebrovascular disease. We investigated whether age modifies the association between the *NOTCH3* p.R544C variant and ischemic stroke risk at the population level.

We integrated data from the Taiwan Biobank (TWB, 2008-2020) with the National Health Insurance Research Database (NHIRD, 2000-2020). Among 112,500 participants, 8,588 ischemic stroke cases were identified. Participants were classified as wild-type (GG) or p.R544C carriers (AG+AA). Logistic regression models were used to estimate stroke risk after adjusting for demographic characteristics, vascular comorbidities, and lifestyle factors. Effect modification by age was evaluated using interaction terms and age-genotype-stratified analyses.

Overall, p.R544C carriers exhibited a higher prevalence of ischemic stroke than noncarriers (10.37% vs. 7.61%). The p.R544C variant was associated with higher odds of ischemic stroke (adjusted odds ratio [OR], 1.54; 95% confidence interval [CI], 1.24-1.90). Age was a strong determinant of stroke risk, and a significant interaction between age and genotype was observed (p for interaction = 0.02), indicating that the effect of age on ischemic stroke risk varied by genotype. In the joint age-genotype model, the highest odds of ischemic stroke were observed among AG+AA carriers aged 51-60 years (OR 7.07, 95% CI 5.08-9.82) and 61-70 years (OR 11.40, 95% CI 8.02-16.19), relative to 30-40-year-old GG carriers.

These findings indicate that the association between the *NOTCH3* p.R544C variant and ischemic stroke risk is age-dependent. The progressively stronger genetic effect observed in later adulthood underscores the importance of incorporating age-specific genetic susceptibility into population-based stroke risk stratification and neurogenetic epidemiology.

## Introduction

Ischemic stroke accounts for the majority of cerebrovascular events worldwide and is shaped by both common polygenic susceptibility and rare monogenic determinants 1, 2. Among monogenic causes, NOTCH3 variants have attracted particular attention because their phenotypic spectrum extends beyond classical CADASIL to include milder and incompletely penetrant forms of cerebral small-vessel disease 3, 4. Although CADASIL was historically described as a fully penetrant autosomal dominant disorder, recent evidence indicates that many NOTCH3 variant carriers never develop the full syndrome, suggesting that penetrance is substantially incomplete at the population level 4-6. Recent population-scale sequencing has shown that cysteine-altering NOTCH3 variants are substantially more frequent than historically assumed and contribute to a continuum of small-vessel cerebrovascular disease, from asymptomatic carriers to typical CADASIL 4, 7, 8.

Within this expanded clinical spectrum, the EGFr-domain risk-stratification framework proposed by Hack and colleagues classifies cysteine-altering NOTCH3 variants into high-, moderate-, and low-risk tiers according to the position of the affected EGFr domain and observed penetrance 9. The p.Arg544Cys substitution (rs201118034) is predicted to lie between EGFr domains 13 and 14 (annotated as EGFr 13.5) and is therefore not part of the high-risk EGFr domains 1-6, 8, 11, or 26 defined in the three-tiered risk framework, but falls instead within the broader EGFr 7-34 group that is, on average, associated with later onset and milder NOTCH3-SVD than EGFr 1-6 variants. In this context, variants such as p.Arg544Cys are expected, on average, to show lower penetrance and later or milder clinical presentations than canonical high-risk CADASIL mutations in EGFr 1-6, although substantial variability between individuals remains. Although uncommon globally, p.R544C reaches an appreciable allele frequency in East Asian populations, particularly in Taiwan 10-12, making it a tractable variant for evaluating moderate-impact NOTCH3 alleles in unselected community cohorts rather than in CADASIL referral series.

Age is the strongest non-modifiable determinant of ischemic stroke and may also reshape the apparent effect of genetic predisposition across the life course 13-15. For NOTCH3-related disease, cross-sectional and cohort data indicate that the clinical expression of cysteine-altering variants intensifies with advancing age, partly through accumulation of vascular comorbidities and white-matter injury 6, 16, 17. Lacunar-stroke and small-vessel cohorts have likewise reported that cysteine-altering NOTCH3 variants—including p.R544C in Taiwanese patients with small-vessel occlusion stroke—are enriched compared with the general population 12, 18 and large biobank studies have shown that the predictive weight of stroke-associated genetic variants varies with the age window under examination 19, 20. However, whether this age dependence applies specifically to moderate-risk alleles such as p.R544C at the population level has not been formally tested.

We previously reported, using the TWB linked to the NHIRD, that the rs201118034 AG+AA genotype is associated with increased risks of ischemic and hemorrhagic stroke but not dementia 21. That analysis treated rs201118034 as a single, age-adjusted exposure across the full adult cohort and did not examine whether the magnitude of the genetic effect differs across life stages. However, the prior literature summarized above suggests that age may not simply confound the p.R544C-stroke relationship but may instead modify the variant's clinical impact. Distinguishing these two roles requires explicit interaction modelling and age-stratified estimation, which were outside the scope of our earlier report.

Therefore, the present study extends but does not replicate our previous analysis. Using the same TWB-NHIRD linkage, we test the prespecified hypothesis that age modifies the association between the NOTCH3 p.R544C (rs201118034) variant and ischemic stroke risk, and we quantify the joint effect of age and genotype on stroke odds in a population-based Taiwanese cohort. By focusing exclusively on ischemic stroke and age as a biologically motivated effect modifier, this analysis addresses a question that our earlier paper raised but did not answer, and informs whether age-specific interpretation of carrier status is warranted in clinical and public-health risk assessment.

## Methods

### Data sources and study design

This study integrated genetic and phenotypic data from the TWB (2008-2020), with longitudinal medical records from the NHIRD (2000-2020). Individual-level linkage was performed using anonymized identifiers to preserve privacy.

### Outcome definition and covariates

Ischemic stroke cases were ascertained from the NHIRD using validated diagnostic criteria based on the International Classification of Diseases, Ninth Revision, Clinical Modification (ICD-9-CM: 433-437) and Tenth Revision (ICD-10: I63, I65-I67). Cases were defined as individuals with at least one inpatient admission or two outpatient visits carrying relevant codes during the observation period. Major vascular comorbidities—including hypertension, diabetes mellitus, hyperlipidemia, and atrial fibrillation—were identified using standard ICD coding algorithms and incorporated as covariates.

### Study sample

Among the 132,720 TWB participants initially enrolled, individuals were excluded if their genotyping data were unavailable (n = 18,329), questionnaire information was incomplete (n = 158), or they had a history of non-ischemic stroke or dementia (n = 1,733). The resulting analytic cohort included 112,500 participants, comprising 8,588 ischemic stroke cases and 103,912 controls. This sample forms the basis for evaluating gene-age interaction effects rather than replicating primary association estimates reported previously.

### Genotyping and variant definition

Genetic data were generated using TWBv1 and TWBv2 arrays. The rs201118034 variant in the NOTCH3 gene—corresponding to the p.R544C missense mutation—was the primary exposure of interest. Quality control measures confirmed a missing data rate below 0.1%, a Hardy-Weinberg equilibrium p-value of 1, and a minor allele frequency (MAF) of 0.0047. Given the low MAF, heterozygous and homozygous (AG+AA) individuals were combined into a single group and compared against wild-type (GG) individuals.

### Exposure and covariate assessment

Baseline demographic and lifestyle characteristics were obtained from TWB questionnaires. Variables included age, sex, body mass index (BMI), smoking, alcohol intake, and physical activity. Behavioral factors were defined using standardized thresholds for habitual exposure, as previously defined 21. For interaction analysis, age was categorized into four predefined strata (30-40, 41-50, 51-60, and 61-70 years), enabling assessment of potential non-uniform genetic effects across life stages. BMI was similarly categorized for adjustment and subgroup comparisons.

### Statistical analysis

Multivariable logistic regression models were applied to estimate the association between rs201118034 and ischemic stroke while adjusting for demographic, clinical, and lifestyle factors, including age, sex, smoking, alcohol consumption, physical activity, body mass index, diabetes, hypertension, hyperlipidemia, and atrial fibrillation. Age was categorized into four groups (30-40, 41-50, 51-60, and 61-70 years) and coded as an ordinal variable from 0 to 3, with 30-40 years serving as the baseline category. The results of the multivariable logistic regression model are presented as ORs with 95% CIs. To assess whether age modified the association between rs201118034 genotype and ischemic stroke risk, an interaction term between genotype and age was subsequently incorporated into the multivariable logistic regression model. Additional age-stratified analyses were conducted to further characterize the association between rs201118034 genotype and ischemic stroke risk across age groups. Statistical significance was defined as a two-sided p-value < 0.05.

## Results

Table 1 summarizes the demographic and clinical characteristics of the study population, stratified by rs201118034 genotype (GG vs. AG+AA). Among the 112,500 participants, 1061 (0.94%) carried at least one A allele (AG+AA), whereas 111,439 (99.06%) were homozygous for the G allele (GG). Stroke prevalence was higher among AG+AA carriers (10.37% vs. 7.61%; p = 0.001). Age distribution and sex proportions were similar between genotypes, with no significant differences. AG+AA carriers exhibited a lower prevalence of smoking (17.25% vs. 19.85%, p = 0.030) but a higher proportion of BMI ≥ 27 kg/m² (23.75% vs. 20.28%, p = 0.049). Other factors, such as alcohol consumption, physical activity, diabetes, hypertension, hyperlipidemia, and atrial fibrillation, showed no significant genotype differences.

The results of the multivariable logistic regression analysis are presented in Table 2. The AG+AA genotype of rs201118034 was significantly associated with increased odds of ischemic stroke compared with the GG genotype (OR: 1.54, 95% CI: 1.24-1.90, p < 0.001). Age was a strong determinant, with the odds of ischemic stroke increasing across older age groups: 41-50 years (OR: 1.99, 95% CI: 1.76-2.24), 51-60 years (OR: 3.80, 95% CI: 3.40-4.25), and 61-70 years (OR: 6.63, 95% CI: 5.92-7.43) compared with the 30-40-year age group. Other significant predictors included diabetes (OR: 1.30, 95% CI: 1.23-1.37, p < 0.001), hypertension (OR: 2.34, 95% CI: 2.22-2.47, p < 0.001), hyperlipidemia (OR: 2.06, 95% CI: 1.94-2.18, p < 0.001), and atrial fibrillation (OR: 2.09, 95% CI: 1.84-2.39, p < 0.001). In contrast, smoking, alcohol use, BMI, and physical activity demonstrated weaker or non-significant associations. These results highlight age, genotype, and clinical comorbidities as critical stroke risk factors.

Table 3 presents the interaction analysis between rs201118034 genotype and age on ischemic stroke risk using multivariable logistic regression. In the interaction model, the main effect of rs201118034 (AG+AA vs. GG) represented the genotype-specific association with ischemic stroke risk at the baseline age group of 30-40 years (OR = 0.74, 95% CI: 0.39-1.42, p = 0.368), rather than a marginal association across all age groups. The genotype-by-age interaction was statistically significant (β = 0.33, OR = 1.40, 95% CI: 1.06-1.83, p = 0.020), suggesting that the association between rs201118034 genotype and ischemic stroke risk increased progressively across the four ordinal age groups.

Table 4 presents the association between age and ischemic stroke risk, stratified by rs201118034 genotype (GG vs. AG+AA), using multiple logistic regression. Age significantly influences ischemic stroke risk in both genotype groups. Among GG carriers, the ORs increase progressively with age: 41-50 years (OR: 1.99, 95% CI: 1.76-2.24), 51-60 years (OR: 3.76, 95% CI: 3.36-4.21), and 61-70 years (OR: 6.58, 95% CI: 5.87-7.37), all significant (p < 0.001). AG+AA carriers show even greater risk escalation in older age groups: 51-60 years (OR: 10.54, 95% CI: 2.44-45.49, p = 0.002) and 61-70 years (OR: 15.15, 95% CI: 3.43-67.02, p < 0.001).

Table 5 reports the association for combined age-genotype categories relative to the single reference group (30-40, GG) in the same multivariable framework. Among individuals with the GG genotype, the odds of ischemic stroke rose steadily from ages 41-50 (OR: 1.98, 95% CI: 1.76-2.24) to 61-70 (OR: 6.55, 95% CI: 5.84-7.34). A similar but more pronounced gradient was observed among AG+AA carriers, with markedly elevated risks in the 51-60 (OR: 7.07, 95% CI: 5.08-9.82) and 61-70 (OR: 11.4, 95% CI: 8.02-16.19) age groups. Notably, the joint presence of older age and the AG+AA genotype was associated with markedly higher odds of ischemic stroke compared with GG carriers within the same age strata.

## Discussion

This study demonstrates that the NOTCH3 rs201118034 variant is independently associated with higher odds of ischemic stroke and that this association varies according to age. Across multiple analytic approaches, age emerged as a strong determinant of stroke risk, with progressively increasing odds observed in older age groups. Importantly, a significant interaction between age and rs201118034 genotype indicates that the effect of age on ischemic stroke risk varied by genotype. Within the same age strata, the ORs for AG+AA carriers were generally higher than those for GG carriers among older individuals, particularly in the 51-70-year age groups, whereas lower or comparable ORs were observed for AG+AA carriers in the younger age strata (30-40 and 41-50 years) in the joint model.

Combined age-genotype models demonstrated that the highest odds of ischemic stroke occurred among older AG+AA carriers, suggesting that the association between genotype and ischemic stroke may vary with age. Within the genotype-stratified analysis, 41-50-year-old AG+AA carriers had higher odds of ischemic stroke than the 30-40-year-old AG+AA reference group (OR 2.10, 95% CI 0.42-10.53). However, the wide confidence interval and the small number of AG+AA cases in this age stratum (n = 7) preclude definitive conclusions regarding the magnitude of this association. Importantly, this stratum-specific estimate should be distinguished from the joint-model estimate in Table 5, where the 41-50-year AG+AA group was compared with the 30-40-year GG reference group (OR 1.37, 95% CI 0.64-2.94). The p.R544C variant is characterized by incomplete penetrance and age-dependent clinical expression; at younger ages, the cumulative vascular burden associated with genetic susceptibility and vascular risk factors may not yet be sufficient to manifest as clinically detectable stroke. This interpretation is consistent with the known biology of moderate-risk NOTCH3 variants, in which white matter lesion accumulation and small-vessel injury progress gradually over decades 9, 16, 17. The markedly elevated ORs observed in the 51-60 and 61-70 age strata among AG+AA carriers support the hypothesis that the clinical impact of this variant becomes increasingly evident only in later adulthood.

While the underlying mechanisms cannot be determined from this study, the elevated stroke risk observed in p.R544C carriers is consistent with prior Taiwanese case-control studies, which reported approximately 3.4-fold and 11-fold increases in overall and small-vessel occlusion stroke risk, respectively 12. Similarly, large-scale population studies, including the UK Biobank analyses, have shown that cysteine-altering NOTCH3 variants are associated with increased risks of stroke, vascular dementia, and MRI markers of small-vessel disease, supporting a broader contribution of NOTCH3 to “sporadic” stroke 4, 6.

The strong effect of age and the amplified risk observed in older AG+AA individuals are consistent with the known phenotypic heterogeneity of NOTCH3 cysteine-altering variants, which range from asymptomatic carriers to individuals with classical CADASIL 4, 5, 9. A community-based cohort study has demonstrated that many carriers remain free of stroke or significant MRI abnormalities even in later life, consistent with the incomplete clinical expression reported in a previous study and the large proportion of AG+AA carriers who remained stroke-free in our cohort 4.

Notably, male sex was associated with modestly lower odds of ischemic stroke compared with female sex in this cohort (OR 0.94, 95% CI 0.89-0.99, p = 0.034). This finding appears to contrast with the generally higher stroke incidence reported in men in population-based epidemiological studies. This pattern may reflect the demographic composition of the Taiwan Biobank, in which women constitute approximately 64% of participants, and healthy volunteer bias may differentially affect the male subgroup. Alternatively, the sex difference observed here may partly reflect the age distribution of the cohort, as TWB participants are predominantly in mid-adulthood, a life stage at which the male excess in stroke risk is less pronounced than in older age groups. Importantly, the sex-by-genotype interaction term was not statistically significant (OR 1.16, 95% CI 0.67-2.00, p = 0.595), indicating the association between sex and ischemic stroke did not significantly differ by genotype. The sex distribution was also similar between GG and AG+AA carriers (male: 35.95% vs. 34.78%, p = 0.554), further supporting that sex did not act as a differential confounder in the primary analysis. These considerations underscore the importance of interpreting sex-specific estimates within the context of the study design and sampling frame.

Other studies have indicated that the impact of stroke-associated genetic variants may vary by age at onset *19* and that the interplay between genetic predisposition and acquired risk factors varies with advancing age 20*.* The significant age-NOTCH3 interaction identified in the present study extends this concept to the specific moderate-risk p.R544C variant.

From a clinical perspective, our findings support NOTCH3 p.R544C as an important population-level risk factor in East Asian populations, and further suggest that age-stratified risk assessment may be necessary when interpreting carrier status 11, 22. The contrast between elevated ORs in older AG+AA individuals and the substantial proportion of carriers who remained free of ischemic stroke highlights the likely influence of additional modifiers, including specific EGFr domain involvement, coexisting vascular risk factors, and broader genetic background 5, 9.

Although age and the rs201118034 genotype are non-modifiable, evidence from prior studies suggests that modifiable vascular risk factors may influence disease expression in NOTCH3-related small vessel disease, including CADASIL. In particular, hypertension has been consistently associated with increased stroke risk and disease severity in CADASIL populations. A recent meta-analysis reported that hypertension was associated with an approximately 2.7-fold increase in stroke risk in CADASIL, underscoring its role as a key modifiable determinant of clinical outcomes 22. In addition, observational studies have shown that conventional vascular risk factors—especially hypertension and smoking—are linked to earlier stroke onset and greater radiological burden, including progression of white matter lesions 23, 24. These findings suggest that, despite the genetic basis of NOTCH3-related disease, vascular risk factor control may influence clinical outcomes.

However, management implications should be interpreted cautiously. Although this study included environmental variables such as smoking and alcohol consumption, their interactions with the rs201118034 genotype were not formally examined, as the primary focus was on the age-genotype interaction. Accordingly, while our findings do not directly establish gene-environment interactions, the observed age-dependent increase in stroke risk among carriers is consistent with the hypothesis that cumulative vascular stress may exacerbate underlying genetic susceptibility. Therefore, optimal management of modifiable risk factors—particularly blood pressure—remains a reasonable strategy in high-risk individuals, although this recommendation is supported by external evidence rather than directly demonstrated in the present analysis.

Future research should expand on these findings by investigating additional environmental and lifestyle modifiers, such as diet, air pollution, and socioeconomic factors, to provide a more comprehensive understanding of stroke risk in genetically susceptible populations.

This study is not without limitations. First, environmental and lifestyle factors were included as covariates but not evaluated as effect modifiers, limiting insight into potential gene-environment interactions. Second, age was categorized into predefined groups, which may have obscured more granular trends or nonlinear relationships. While treating age as a continuous variable or employing finer age group stratifications could offer more detailed characterization, over-stratification may reduce statistical stability due to smaller subgroup sample sizes. Third, the Taiwan Biobank recruited predominantly healthy volunteers, with women comprising approximately 64% of participants. This sampling frame may introduce selection bias and limit the generalizability of sex-specific estimates to the general population. Finally, the very small number of homozygous AA carriers in this population-based biobank precluded a dedicated subgroup analysis, and future, larger studies are needed to address the association between AA homozygosity and ischemic stroke.

## Conclusion

This study provides population-based evidence that the NOTCH3 p.R544C variant is associated with increased odds of ischemic stroke and that its association varies across age groups. A significant interaction between age and rs201118034 genotype indicates that the effect of age on ischemic stroke risk differed according to genotype, with substantially higher odds observed among older AG+AA carriers. These findings are consistent with a model in which age-related vascular changes and monogenic small-vessel susceptibility jointly contribute to stroke risk. The progressively stronger genetic effect observed in later adulthood suggests that the clinical relevance of NOTCH3-related susceptibility becomes increasingly evident with aging. Collectively, these results underscore the importance of integrating genetic information with age and vascular risk profiles to improve risk stratification and inform future precision prevention strategies for cerebrovascular disease.

## Figures and Tables

**Table 1:** Basic demographic features stratified by genotype (GG vs. AG+AA)

	GG		AG+AA		
**Features**	N	(%)	N	(%)	**p-value**
Ischemic stroke					0.001
No	102,961	(92.39)	951	(89.63)	
Yes	8478	(7.61)	110	(10.37)	
Age (yrs)					0.289
30-40	28,143	(25.25)	288	(27.14)	
41-50	26,980	(24.21)	266	(25.07)	
51-60	34,038	(30.54)	319	(30.07)	
61-70	22,278	(19.99)	188	(17.72)	
Sex					0.554
Female	71,382	(64.05)	692	(65.22)	
Male	40,057	(35.95)	369	(34.78)	
Smoking					0.030
No	89,320	(80.15)	878	(82.75)	
Yes	22119	(19.85)	183	(17.25)	
Alcohol consumption					0.355
No	102,032	(91.56)	979	(92.27)	
Yes	9407	(8.44)	82	(7.73)	
Physical activity					0.552
No	66,429	(59.61)	624	(58.81)	
Yes	45,010	(40.39)	437	(41.19)	
Body Mass Index, kg/m^2^					0.049
<18.5	3681	(3.30)	30	(2.83)	
18.5-24	54,960	(49.32)	513	(48.35)	
24-27	30,195	(27.10)	266	(25.07)	
≥27	22,603	(20.28)	252	(23.75)	
Diabetes					0.693
No	91,043	(81.70)	865	(81.53)	
Yes	20,396	(18.30)	196	(18.47)	
Hypertension					0.465
No	79,192	(71.06)	769	(72.48)	
Yes	32,247	(28.94)	292	(27.52)	
Hyperlipidemia					0.185
No	66,402	(59.59)	614	(57.87)	
Yes	45,037	(40.41)	447	(42.13)	
Atrial fibrillation					0.425
No	110,139	(98.83)	1051	(99.06)	
Yes	1300	(1.17)	10	(0.94)	

**Note:** N, number of participants; %, percentage; reference SNP identification number. AG+AA and GG, rs201118034 genotypes

**Table 2:** Multivariable logistic regression analysis of covariates associated with ischemic stroke risk

Variables	OR	(95% CI)	p-value
rs201118034. (ref: GG)			
AG+AA	1.54	(1.24-1.90)	<0.001
Age (ref: 30-40)			
41-50	1.99	(1.76-2.24)	<0.001
51-60	3.80	(3.40-4.25)	<0.001
61-70	6.63	(5.92-7.43)	<0.001
Sex (ref: Female)			
Male	0.94	(0.89-0.99)	0.034
Smoking (ref: No)			
Yes	1.06	(0.99-1.14)	0.079
Alcohol consumption (ref: No)			
Yes	1.05	(0.97-1.14)	0.246
Physical activity (ref: No)			
Yes	1.03	(0.98-1.08)	0.187
BMI (ref: 18.5-24)			
<18.5	1.20	(1.03-1.41)	0.023
24-27	1.04	(0.98-1.10)	0.209
≥27	1.02	(0.95-1.08)	0.652
Diabetes (ref: No)			
Yes	1.30	(1.23-1.37)	<0.001
Hypertension (ref: No)			
Yes	2.34	(2.22-2.47)	<0.001
Hyperlipidemia (ref: No)			
Yes	2.06	(1.94-2.18)	<0.001
Atrial fibrillation (ref: No)			
Yes	2.09	(1.84-2.39)	<0.001

**Note:** OR, odds ratio; CI, confidence interval; ref, reference category; BMI, body mass index; AG+AA and GG, rs201118034 genotypes.

**Table 3:** Interaction analysis of rs201118034 genotype and age on ischemic stroke risk using multiple logistic regression

Variables	BETA	OR	(95% CI)	p-value
rs201118034 * Age	0.33	1.40	(1.06-1.83)	0.020
rs201118034 (ref: GG)				
AG+AA	-0.30	0.74	(0.39-1.42)	0.368
Age (ref: 30-40)				
41-50	0.68	1.98	(1.75-2.23)	<0.001
51-60	1.33	3.77	(3.37-4.22)	<0.001
61-70	1.88	6.55	(5.85-7.34)	<0.001
Sex (ref: Female)				
Male	-0.06	0.94	(0.89-0.99)	0.034
Smoking (ref: No)				
Yes	0.06	1.06	(0.99-1.14)	0.081
Alcohol consumption (ref: No)				
Yes	0.05	1.05	(0.97-1.14)	0.247
Physical activity (ref: No)				
Yes	0.03	1.03	(0.98-1.08)	0.189
BMI (ref: 18.5-24)				
<18.5	0.18	1.20	(1.03-1.41)	0.023
24-27	0.04	1.04	(0.98-1.10)	0.210
≥27	0.01	1.01	(0.95-1.08)	0.656
Diabetes (ref: No)				
Yes	0.26	1.30	(1.23-1.37)	<0.001
Hypertension (ref: No)				
Yes	0.85	2.34	(2.22-2.47)	<0.001
Hyperlipidemia (ref: No)				
Yes	0.72	2.06	(1.94-2.18)	<0.001
Atrial fibrillation (ref: No)				
Yes	0.74	2.09	(1.84-2.39)	<0.001

**Note:** OR, odds ratio; CI, confidence interval; ref, reference category; BMI, body mass index; AG+AA and GG, rs201118034 genotypes.

**Table 4:** Association between age and ischemic stroke stratified by rs201118034 genotype (GG vs. AG+AA) using multiple logistic regression

	GG			AG+AA		
Variables	OR	(95% CI)	p-value	OR	(95% CI)	p-value
Age (ref: 30-40)						
41-50	1.99	(1.76-2.24)	<0.001	2.10	(0.42-10.53)	0.369
51-60	3.76	(3.36-4.21)	<0.001	10.54	(2.44-45.49)	0.002
61-70	6.58	(5.87-7.37)	<0.001	15.15	(3.43-67.02)	<0.001
Sex (ref: Female)						
Male	0.94	(0.88-0.99)	0.028	1.16	(0.67-2.00)	0.595
Smoking (ref: No)						
Yes	1.06	(0.99-1.14)	0.086	1.24	(0.65-2.39)	0.512
Alcohol consumption (ref: No)						
Yes	1.05	(0.96-1.14)	0.278	1.40	(0.65-3.03)	0.389
Physical activity (ref: No)						
Yes	1.03	(0.98-1.09)	0.187	1.05	(0.65-1.69)	0.852
BMI (ref: 18.5-24)						
<18.5	1.19	(1.02-1.40)	0.029	3.12	(0.63-15.46)	0.164
24-27	1.04	(0.98-1.10)	0.211	0.99	(0.56-1.77)	0.980
≥27	1.02	(0.96-1.08)	0.606	0.70	(0.38-1.29)	0.252
Diabetes (ref: No)						
Yes	1.30	(1.23-1.37)	<0.001	1.19	(0.72-1.95)	0.499
Hypertension (ref: No)						
Yes	2.33	(2.20-2.45)	<0.001	4.57	(2.69-7.76)	<0.001
Hyperlipidemia (ref: No)						
Yes	2.05	(1.93-2.17)	<0.001	3.93	(2.08-7.41)	<0.001
Atrial fibrillation (ref: No)						
Yes	2.09	(1.83-2.39)	<0.001	2.25	(0.54-9.39)	0.267

**Note:** OR, odds ratio; CI, confidence interval; ref, reference category; BMI, body mass index; AG+AA and GG, rs201118034 genotypes.

**Table 5:** Joint risk pattern across age and genotype categories using multiple logistic regression

Age-genotype categories	OR	(95% CI)	p-value
Age and rs201118034 genotype (ref: 30-40, GG)			
30-40, AG+AA	0.49	(0.12-1.99)	0.319
41-50, GG	1.98	(1.76-2.24)	<0.001
41-50, AG+AA	1.37	(0.64-2.94)	0.423
51-60, GG	3.75	(3.35-4.19)	<0.001
51-60, AG+AA	7.07	(5.08-9.82)	<0.001
61-70, GG	6.55	(5.84-7.34)	<0.001
61-70, AG+AA	11.4	(8.02-16.19)	<0.001

**Note:** OR, odds ratio; CI, confidence interval; ref, reference category; BMI, body mass index; AG+AA and GG, rs201118034 genotypes. Adjusted for gender, smoking status, alcohol consumption, physical activity, BMI, diabetes, hypertension, hyperlipidemia, and atrial fibrillation.

## Data Availability

The data that support the findings of this study are available from the Taiwan Biobank data source. However, restrictions apply to the availability of these data, which were used under license for the current study, and so are not publicly available. Data are, however, available from the authors upon reasonable request and with permission of Taiwan Biobank.
